# Supplementary material for: Computational imaging of moving objects obscured by a random corridor via speckle correlations
Source: Nat Commun. 2022 Jul 14;13:4081. doi: 10.1038/s41467-022-31669-7 (PMC9283427; doi:10.1038/s41467-022-31669-7)
Supplement: Supplementary file 1 — Supplementary Information [file 41467_2022_31669_MOESM1_ESM.pdf]

Supplementary Information for

Computational imaging of moving objects obscured by a random corridor via speckle correlations

Tian Shi[1,2], Liangsheng Li[2,*], He Cai[2], Xianli Zhu[2], Qingfan Shi[1], and Ning Zheng[1,*]

[1]School of Physics, Beijing Institute of Technology, Beijing, China
[2]Science and Technology on Electromagnetic Scattering Laboratory, Beijing, China
* ningzheng@bit.edu.cn; liliangshengbititp@163.com

Supplementary Note 1:

Imaging principle of RSESIC method

In the paper, the cross-correlation of speckle intensity images $C_{\mathrm{rd}}(\Delta\boldsymbol{\rho})$ calculated by the RSESIC method is approximately equivalent to the transmitted field autocorrelation of a hidden object $C_{\mathrm{ho}}(\Delta\boldsymbol{\rho})$ under the condition of multiple scattering. Hidden objects in random corridors can be imaged with the approximation. The approximate relationship can be expressed as,

$$C_{\mathrm{rd}}(\Delta\boldsymbol{\rho}) \approx C_{\mathrm{ho}}(\Delta\boldsymbol{\rho}) \tag{1}$$

This relationship can be derived from the definition of $C_{\mathrm{rd}}(\Delta\boldsymbol{\rho})$ based on three assumptions. $C_{\mathrm{rd}}(\Delta\boldsymbol{\rho})$ is defined as,

$$C_{\mathrm{rd}}(\Delta\mathbf{r}) = \left\langle \frac{\left[I_{\mathrm{rd}}(\mathbf{r}_{\mathrm{c}};\mathbf{r}_{\mathrm{o}}) - \left\langle I_{\mathrm{rd}}(\mathbf{r}_{\mathrm{c}};\mathbf{r}_{\mathrm{o}})\right\rangle_{\mathbf{r}_{\mathrm{c}}}\right]\left[I_{\mathrm{rd}}(\mathbf{r}_{\mathrm{c}};\mathbf{r}_{\mathrm{o}}+\Delta\boldsymbol{\rho}) - \left\langle I_{\mathrm{rd}}(\mathbf{r}_{\mathrm{c}};\mathbf{r}_{\mathrm{o}}+\Delta\boldsymbol{\rho})\right\rangle_{\mathbf{r}_{\mathrm{c}}}\right]}{\sigma_{\mathrm{rd}}(\mathbf{r}_{\mathrm{o}})\sigma_{\mathrm{rd}}(\mathbf{r}_{\mathrm{o}}+\Delta\boldsymbol{\rho})} \right\rangle_{\mathbf{r}_{\mathrm{c}},\mathbf{r}_{\mathrm{o}}} \tag{2}$$

The ensemble average $\langle \ldots \rangle_{\mathbf{r}_{\mathrm{o}}}$ is used to increase the overall average times. Therefore, $C_{\mathrm{rd}}(\Delta\boldsymbol{\rho})$ is expressed as,

$$C_{\mathrm{rd}}(\Delta\mathbf{r}) = \frac{\left\langle\left[I_{\mathrm{rd}}(\mathbf{r}_{\mathrm{c}};\mathbf{r}_{\mathrm{o}}) - \left\langle I_{\mathrm{rd}}(\mathbf{r}_{\mathrm{c}};\mathbf{r}_{\mathrm{o}})\right\rangle_{\mathbf{r}_{\mathrm{c}}}\right]\left[I_{\mathrm{rd}}(\mathbf{r}_{\mathrm{c}};\mathbf{r}_{\mathrm{o}}+\Delta\boldsymbol{\rho}) - \left\langle I_{\mathrm{rd}}(\mathbf{r}_{\mathrm{c}};\mathbf{r}_{\mathrm{o}}+\Delta\boldsymbol{\rho})\right\rangle_{\mathbf{r}_{\mathrm{c}}}\right]\right\rangle_{\mathbf{r}_{\mathrm{c}}}}{\sigma_{\mathrm{rd}}(\mathbf{r}_{\mathrm{o}})\sigma_{\mathrm{rd}}(\mathbf{r}_{\mathrm{o}}+\Delta\boldsymbol{\rho})} \tag{3}$$

The numerator is expanded in formula (3) to obtain,

$$C_{\mathrm{rd}}(\Delta\mathbf{r})=\frac{\langle I_{\mathrm{rd}}(\mathbf{r}_\mathrm{c};\mathbf{r}_\mathrm{o})I_{\mathrm{rd}}(\mathbf{r}_\mathrm{c};\mathbf{r}_\mathrm{o}+\Delta\boldsymbol{\rho})\rangle_{\mathbf{r}_\mathrm{c}}-\langle I_{\mathrm{rd}}(\mathbf{r}_\mathrm{c};\mathbf{r}_\mathrm{o})\rangle_{\mathbf{r}_\mathrm{c}}\langle I_{\mathrm{rd}}(\mathbf{r}_\mathrm{c};\mathbf{r}_\mathrm{o}+\Delta\boldsymbol{\rho})\rangle_{\mathbf{r}_\mathrm{c}}}{\sigma_{\mathrm{rd}}(\mathbf{r}_\mathrm{o})\sigma_{\mathrm{rd}}(\mathbf{r}_\mathrm{o}+\Delta\boldsymbol{\rho})} \tag{4}$$

The reduced electric field of scattered light is denoted as E_{rd}, and then the reduced intensity of scattered light can be expanded as $I_{\mathrm{rd}}=E_{\mathrm{rd}}E_{\mathrm{rd}}^*$.

Assumption 1: E_{rd} follows a Gaussian distribution with zero mean. Apply a moment theorem for complex Gaussian [1], and the first term of the numerator in Eq(4) can be expanded as,

$$\begin{aligned}
&\langle I_{\mathrm{rd}}(\mathbf{r}_\mathrm{c};\mathbf{r}_\mathrm{o})I_{\mathrm{rd}}(\mathbf{r}_\mathrm{c};\mathbf{r}_\mathrm{o}+\Delta\boldsymbol{\rho})\rangle_{\mathbf{r}_\mathrm{c}}\\
=&\ \langle E_{\mathrm{rd}}(\mathbf{r}_\mathrm{c};\mathbf{r}_\mathrm{o})E^*_{\mathrm{rd}}(\mathbf{r}_\mathrm{c};\mathbf{r}_\mathrm{o})E_{\mathrm{rd}}(\mathbf{r}_\mathrm{c};\mathbf{r}_\mathrm{o}+\Delta\boldsymbol{\rho})E^*_{\mathrm{rd}}(\mathbf{r}_\mathrm{c};\mathbf{r}_\mathrm{o}+\Delta\boldsymbol{\rho})\rangle_{\mathbf{r}_\mathrm{c}}\\
=&\ \left|\langle E_{\mathrm{rd}}(\mathbf{r}_\mathrm{c};\mathbf{r}_\mathrm{o})E^*_{\mathrm{rd}}(\mathbf{r}_\mathrm{c};\mathbf{r}_\mathrm{o}+\Delta\boldsymbol{\rho})\rangle_{\mathbf{r}_\mathrm{c}}\right|^2+\langle |E_{\mathrm{rd}}(\mathbf{r}_\mathrm{c};\mathbf{r}_\mathrm{o})|^2\rangle_{\mathbf{r}_\mathrm{c}}\langle |E_{\mathrm{rd}}(\mathbf{r}_\mathrm{c};\mathbf{r}_\mathrm{o}+\Delta\boldsymbol{\rho})|^2\rangle_{\mathbf{r}_\mathrm{c}}\\
=&\ \left|\langle E_{\mathrm{rd}}(\mathbf{r}_\mathrm{c};\mathbf{r}_\mathrm{o})E^*_{\mathrm{rd}}(\mathbf{r}_\mathrm{c};\mathbf{r}_\mathrm{o}+\Delta\boldsymbol{\rho})\rangle_{\mathbf{r}_\mathrm{c}}\right|^2+\langle I_{\mathrm{rd}}(\mathbf{r}_\mathrm{c};\mathbf{r}_\mathrm{o})\rangle_{\mathbf{r}_\mathrm{c}}\langle I_{\mathrm{rd}}(\mathbf{r}_\mathrm{c};\mathbf{r}_\mathrm{o}+\Delta\boldsymbol{\rho})\rangle_{\mathbf{r}_\mathrm{c}}
\end{aligned} \tag{5}$$

At the same time, the denominator in Eq. (4) reads[2],

$$\begin{aligned}
&\sigma_{\mathrm{rd}}(\mathbf{r}_\mathrm{o})\sigma_{\mathrm{rd}}(\mathbf{r}_\mathrm{o}+\Delta\boldsymbol{\rho})\\
=&\ \langle I_{\mathrm{rd}}(\mathbf{r}_\mathrm{c};\mathbf{r}_\mathrm{o})\rangle_{\mathbf{r}_\mathrm{c}}\langle I_{\mathrm{rd}}(\mathbf{r}_\mathrm{c};\mathbf{r}_\mathrm{o}+\Delta\boldsymbol{\rho})\rangle_{\mathbf{r}_\mathrm{c}}\\
=&\ \langle |E_{\mathrm{rd}}(\mathbf{r}_\mathrm{c};\mathbf{r}_\mathrm{o})|^2\rangle_{\mathbf{r}_\mathrm{c}}\langle |E_{\mathrm{rd}}(\mathbf{r}_\mathrm{c};\mathbf{r}_\mathrm{o}+\Delta\boldsymbol{\rho})|^2\rangle_{\mathbf{r}_\mathrm{c}}
\end{aligned} \tag{6}$$

Substituting (5) and (6) into (4), we have,

$$C_{\mathrm{rd}}(\Delta\mathbf{r})=\frac{\langle E_{\mathrm{rd}}(\mathbf{r}_\mathrm{c};\mathbf{r}_\mathrm{o})E^*_{\mathrm{rd}}(\mathbf{r}_\mathrm{c};\mathbf{r}_\mathrm{o}+\Delta\boldsymbol{\rho})\rangle_{\mathbf{r}_\mathrm{c}}}{\langle |E_{\mathrm{rd}}(\mathbf{r}_\mathrm{c};\mathbf{r}_\mathrm{o})|^2\rangle_{\mathbf{r}_\mathrm{c}}}\cdot\frac{\langle E^*_{\mathrm{rd}}(\mathbf{r}_\mathrm{c};\mathbf{r}_\mathrm{o})E_{\mathrm{rd}}(\mathbf{r}_\mathrm{c};\mathbf{r}_\mathrm{o}+\Delta\boldsymbol{\rho})\rangle_{\mathbf{r}_\mathrm{c}}}{\langle |E_{\mathrm{rd}}(\mathbf{r}_\mathrm{c};\mathbf{r}_\mathrm{o}+\Delta\boldsymbol{\rho})|^2\rangle_{\mathbf{r}_\mathrm{c}}} \tag{7}$$

We denote the transmitted field of the hidden object as E_{ho}. Previous studies have shown that E_{rd} is a function of E_{ho} [3],

$$E_{\mathrm{rd}}(\mathbf{r}_\mathrm{c};\mathbf{r}_\mathrm{o})=\int d\mathbf{r}K_{\mathrm{rd}}(\mathbf{r}_\mathrm{c},\mathbf{r})\cdot E_{\mathrm{ho}}(\mathbf{r};\mathbf{r}_\mathrm{o}) \tag{8}$$

$$E_{\mathrm{rd}}(\mathbf{r}_\mathrm{c};\mathbf{r}_\mathrm{o}+\Delta\boldsymbol{\rho})=\int d\mathbf{r}K_{\mathrm{rd}}(\mathbf{r}_\mathrm{c},\mathbf{r})\cdot E_{\mathrm{ho}}(\mathbf{r};\mathbf{r}_\mathrm{o}+\Delta\boldsymbol{\rho}) \tag{9}$$

Here $\mathbf{r}$ represents the position vector in three-dimensional space with random media, $K_{\mathrm{rd}}(\mathbf{r}_\mathrm{c},\mathbf{r})$ represents the subspace reduced generalized propagator (SRGP) from $\mathbf{r}$ to $\mathbf{r}_\mathrm{c}$. Substituting (8) and (9) into (7),

$$\begin{aligned}
&\left\langle E_{\mathrm{rd}}(\mathbf{r}_{\mathrm{c}};\mathbf{r}_{\mathrm{o}})E_{\mathrm{rd}}^{*}(\mathbf{r}_{\mathrm{c}};\mathbf{r}_{\mathrm{o}}+\Delta\boldsymbol{\rho})\right\rangle_{\mathbf{r}_{\mathrm{c}}}\\
=&\left\langle \int E_{\mathrm{ho}}(\mathbf{r}';\mathbf{r}_{\mathrm{o}})K_{\mathrm{rd}}(\mathbf{r}_{\mathrm{c}},\mathbf{r}')d\mathbf{r}'\int E_{\mathrm{ho}}^{*}(\mathbf{r}'';\mathbf{r}_{\mathrm{o}}+\Delta\boldsymbol{\rho})K_{\mathrm{rd}}^{*}(\mathbf{r}_{\mathrm{c}},\mathbf{r}'')d\mathbf{r}''\right\rangle_{\mathbf{r}_{\mathrm{c}}}\\
=&\left\langle \int\int E_{\mathrm{ho}}(\mathbf{r}';\mathbf{r}_{\mathrm{o}})E_{\mathrm{ho}}^{*}(\mathbf{r}'';\mathbf{r}_{\mathrm{o}}+\Delta\boldsymbol{\rho})K_{\mathrm{rd}}(\mathbf{r}_{\mathrm{c}},\mathbf{r}')K_{\mathrm{rd}}^{*}(\mathbf{r}_{\mathrm{c}},\mathbf{r}'')d\mathbf{r}'d\mathbf{r}''\right\rangle_{\mathbf{r}_{\mathrm{c}}}
\end{aligned}\tag{10}$$

Assumption 2: When $\mathbf{r}'\neq\mathbf{r}''$, $\int K_{\mathrm{rd}}(\mathbf{r}_{\mathrm{c}},\mathbf{r}')K_{\mathrm{rd}}^{*}(\mathbf{r}_{\mathrm{c}},\mathbf{r}'')d\mathbf{r}'=0$, namely, SRGP from different spatial locations to $\mathbf{r}_{\mathrm{c}}$ is independent of each other. Therefore, equation (10) can be reduced to,

$$\begin{aligned}
&\left\langle E_{\mathrm{rd}}(\mathbf{r}_{\mathrm{c}};\mathbf{r}_{\mathrm{o}})E_{\mathrm{rd}}^{*}(\mathbf{r}_{\mathrm{c}};\mathbf{r}_{\mathrm{o}}+\Delta\boldsymbol{\rho})\right\rangle_{\mathbf{r}_{\mathrm{c}}}\\
=&\left\langle \int E_{\mathrm{ho}}(\mathbf{r};\mathbf{r}_{\mathrm{o}})E_{\mathrm{ho}}^{*}(\mathbf{r};\mathbf{r}_{\mathrm{o}}+\Delta\boldsymbol{\rho})\left|K_{\mathrm{rd}}(\mathbf{r}_{\mathrm{c}},\mathbf{r})\right|^{2}d\mathbf{r}\right\rangle_{\mathbf{r}_{\mathrm{c}}}\\
=&\int E_{\mathrm{ho}}(\mathbf{r};\mathbf{r}_{\mathrm{o}})E_{\mathrm{ho}}^{*}(\mathbf{r};\mathbf{r}_{\mathrm{o}}+\Delta\boldsymbol{\rho})\left\langle\left|K_{\mathrm{rd}}(\mathbf{r}_{\mathrm{c}},\mathbf{r})\right|^{2}\right\rangle_{\mathbf{r}_{\mathrm{c}}}d\mathbf{r}
\end{aligned}\tag{11}$$

Assumption 3: $P_G=\left\langle\left|K_{\mathrm{rd}}(\mathbf{r}_{\mathrm{c}},\mathbf{r})\right|^{2}\right\rangle_{\mathbf{r}_{\mathrm{c}}}$ is a constant and independent of $\mathbf{r}$ and $\mathbf{r}_{\mathrm{c}}$, then,

$$\left\langle E_{\mathrm{rd}}(\mathbf{r}_{\mathrm{c}};\mathbf{r}_{\mathrm{o}})E_{\mathrm{rd}}^{*}(\mathbf{r}_{\mathrm{c}};\mathbf{r}_{\mathrm{o}}+\Delta\boldsymbol{\rho})\right\rangle_{\mathbf{r}_{\mathrm{c}}}=P_G\int E_{\mathrm{ho}}(\mathbf{r};\mathbf{r}_{\mathrm{o}})E_{\mathrm{ho}}^{*}(\mathbf{r};\mathbf{r}_{\mathrm{o}}+\Delta\boldsymbol{\rho})\mathrm{d}\mathbf{r}\tag{12}$$

Similarly,

$$\left\langle\left|E_{\mathrm{rd}}(\mathbf{r}_{\mathrm{c}};\mathbf{r}_{\mathrm{o}})\right|^{2}\right\rangle_{\mathbf{r}_{\mathrm{c}}}=P_G\int\left|E_{\mathrm{ho}}(\mathbf{r};\mathbf{r}_{\mathrm{o}})\right|^{2}d\mathbf{r}\tag{13}$$

$$\left\langle\left|E_{\mathrm{rd}}(\mathbf{r}_{\mathrm{c}};\mathbf{r}_{\mathrm{o}}+\Delta\boldsymbol{\rho})\right|^{2}\right\rangle_{\mathbf{r}_{\mathrm{c}}}=P_G\int\left|E_{\mathrm{ho}}(\mathbf{r};\mathbf{r}_{\mathrm{o}}+\Delta\boldsymbol{\rho})\right|^{2}d\mathbf{r}\tag{14}$$

Therefore, it can be approximated as,

$$\frac{\left\langle E_{\mathrm{rd}}(\mathbf{r}_{\mathrm{c}};\mathbf{r}_{\mathrm{o}})E_{\mathrm{rd}}^{*}(\mathbf{r}_{\mathrm{c}};\mathbf{r}_{\mathrm{o}}+\Delta\boldsymbol{\rho})\right\rangle_{\mathbf{r}_{\mathrm{c}}}}{\left\langle\left|E_{\mathrm{rd}}(\mathbf{r}_{\mathrm{c}};\mathbf{r}_{\mathrm{o}})\right|^{2}\right\rangle_{\mathbf{r}_{\mathrm{c}}}}\approx\frac{\int E_{\mathrm{ho}}(\mathbf{r};\mathbf{r}_{\mathrm{o}})E_{\mathrm{ho}}^{*}(\mathbf{r};\mathbf{r}_{\mathrm{o}}+\Delta\boldsymbol{\rho})d\mathbf{r}}{\int\left|E_{\mathrm{ho}}(\mathbf{r};\mathbf{r}_{\mathrm{o}})\right|^{2}d\mathbf{r}}\tag{15}$$

We define the autocorrelation of the transmitted field of a hidden object as,

$$C_{\mathrm{ho}}(\Delta\boldsymbol{\rho})=\left|\frac{\int E_{\mathrm{ho}}(\mathbf{r};\mathbf{r}_{\mathrm{o}})E_{\mathrm{ho}}^{*}(\mathbf{r};\mathbf{r}_{\mathrm{o}}+\Delta\boldsymbol{\rho})d\mathbf{r}}{\int\left|E_{\mathrm{ho}}(\mathbf{r};\mathbf{r}_{\mathrm{o}})\right|^{2}d\mathbf{r}}\right|^{2}\tag{16}$$

By substituting Eq. (15), (16) into Eq. (7), eventually we obtain the imaging principle of the

RSESIC method,

$$C_{\mathrm{rd}}(\Delta\mathbf{r}) \approx C_{\mathrm{ho}}(\Delta\boldsymbol{\rho}) = \left| \frac{\int E_{\mathrm{ho}}(\mathbf{r};\mathbf{r}_{\mathrm{o}}) E^{*}_{\mathrm{ho}}(\mathbf{r};\mathbf{r}_{\mathrm{o}}+\Delta\boldsymbol{\rho})\, d\mathbf{r}}{\int \left|E_{\mathrm{ho}}(\mathbf{r};\mathbf{r}_{\mathrm{o}})\right|^{2} d\mathbf{r}} \right|^{2} \tag{17}$$

Assumption 2 is satisfied by strong scattering of random media. The subspace reduction we proposed guarantees that Assumption 3 is satisfied regardless of the shape of random medium. Moreover, subspace reduction also ensures the validity of Assumption 1 for any observation position $\mathbf{r}_{\mathrm{c}}$ and any size of spatial statistical region $\langle ... \rangle_{\mathbf{r}_{\mathrm{c}}}$.

In expression (16), $\int d\mathbf{r}$ represents the integral of three-dimensional space including random medium, $E_{\mathrm{ho}}(\mathbf{r};\mathbf{r}_{\mathrm{o}})$ and $E_{\mathrm{ho}}(\mathbf{r};\mathbf{r}_{\mathrm{o}}+\Delta\boldsymbol{\rho})$ represent the three-dimensional transmitted field illuminating on a hidden object. Therefore,

$$\begin{aligned} &\int E_{\mathrm{ho}}(\mathbf{r};\mathbf{r}_{\mathrm{o}}) E^{*}_{\mathrm{ho}}(\mathbf{r};\mathbf{r}_{\mathrm{o}}+\Delta\boldsymbol{\rho})\, d\mathbf{r} \\ =\ &\iiint E_{\mathrm{ho}}(x,y,z;\mathbf{r}_{\mathrm{o}}) E^{*}_{\mathrm{ho}}(x,y,z;\mathbf{r}_{\mathrm{o}}+\Delta\boldsymbol{\rho})\, dxdydz \end{aligned} \tag{18}$$

When the hidden object is a transmission plate and the integral interval of $\int dz$ is relatively small, $E_{\mathrm{ho}}(x,y,z) \approx E_{\mathrm{ho}}(x,y)$, thus,

$$\begin{aligned} &\int E_{\mathrm{ho}}(\mathbf{r};\mathbf{r}_{\mathrm{o}}) E^{*}_{\mathrm{ho}}(\mathbf{r};\mathbf{r}_{\mathrm{o}}+\Delta\boldsymbol{\rho})\, d\mathbf{r} \\ =\ & b_{z} \cdot \iint E_{\mathrm{ho}}(x,y;\mathbf{r}_{\mathrm{o}}) E^{*}_{\mathrm{ho}}(x,y;\mathbf{r}_{\mathrm{o}}+\Delta\boldsymbol{\rho})\, dxdy \end{aligned} \tag{19}$$

Here $b_{z} = \int dz$ is a constant. Applying the Eq. (19) to the Eq. (16), we have,

$$C_{\mathrm{ho}}(\Delta\boldsymbol{\rho}) = \left| \frac{\iint E_{\mathrm{ho}}(x,y;\mathbf{r}_{\mathrm{o}}) E^{*}_{\mathrm{ho}}(x,y;\mathbf{r}_{\mathrm{o}}+\Delta\boldsymbol{\rho})\, dxdy}{\iint \left|E_{\mathrm{ho}}(x,y;\mathbf{r}_{\mathrm{o}})\right|^{2} dxdy} \right|^{2} \tag{20}$$

We define the reduced dimensionless transmitted field as,

$$\hat{E}_{\mathrm{ho}}(x,y;\mathbf{r}_{\mathrm{o}}) = \frac{E_{\mathrm{ho}}(x,y;\mathbf{r}_{\mathrm{o}})}{\sqrt{\iint \left|E_{\mathrm{ho}}(x,y;\mathbf{r}_{\mathrm{o}})\right|^{2} dxdy}} \tag{21}$$

$$\hat{E}_{\mathrm{ho}}(x,y;\mathbf{r}_{\mathrm{o}}+\Delta\boldsymbol{\rho}) = \frac{E_{\mathrm{ho}}(x,y;\mathbf{r}_{\mathrm{o}}+\Delta\boldsymbol{\rho})}{\sqrt{\iint \left|E_{\mathrm{ho}}(x,y;\mathbf{r}_{\mathrm{o}})\right|^{2} dxdy}} \tag{22}$$

By inserting Eq. (21), (22) to Eq. (20) and applying the autocorrelation theorem [4], we have,

$$\begin{aligned} C_{\mathrm{ho}}(\Delta\boldsymbol{\rho}) &= \left| \iint \hat{E}_{\mathrm{ho}}(x,y;\mathbf{r}_{\mathrm{o}}) \hat{E}^{*}_{\mathrm{ho}}(x,y;\mathbf{r}_{\mathrm{o}}+\Delta\boldsymbol{\rho}) dxdy \right|^{2} \\ &= \left| \mathcal{F}^{-1}\left\{ \left| \mathcal{F}\left\{ \hat{E}_{\mathrm{ho}}(x,y) \right\} \right|^{2} \right\} \right|^{2} \end{aligned} \tag{23}$$

Here $\mathcal{F}$ and $\mathcal{F}^{-1}$ represent Fourier transform and inverse Fourier transform, respectively. Then combining Eq. (23) with (1),

$$\hat{E}_{\mathrm{ho}}(x,y) = \mathcal{F}^{-1}\left\{ \sqrt{\mathcal{F}\left\{ \sqrt{C_{\mathrm{rd}}(\Delta\boldsymbol{\rho})} \cdot e^{i\varphi_1(\Delta\boldsymbol{\rho})} \right\}} \cdot e^{i\varphi_2(\mathbf{k})} \right\} \tag{24}$$

In this equation, $\varphi_1(\Delta\boldsymbol{\rho})$ is the phase of $\iint \hat{E}_{\mathrm{ho}}(x,y;\mathbf{r}_{\mathrm{o}}) \hat{E}^{*}_{\mathrm{ho}}(x,y;\mathbf{r}_{\mathrm{o}}+\Delta\boldsymbol{\rho}) dxdy$. When the hidden object is a transmission plate, we find that the imaginary part of $\iint \hat{E}_{\mathrm{ho}}(x,y;\mathbf{r}_{\mathrm{o}}) \hat{E}^{*}_{\mathrm{ho}}(x,y;\mathbf{r}_{\mathrm{o}}+\Delta\boldsymbol{\rho}) dxdy$ is much smaller than its real part, so $\varphi_1(\Delta\boldsymbol{\rho}) \approx 0$. $\varphi_2(\mathbf{k})$ can be recovered by an iterative phase recovery algorithm[5, 6]. Substituting $\varphi_1(\Delta\boldsymbol{\rho})$, $\varphi_2(\mathbf{k})$ and $C_{\mathrm{rd}}(\Delta\boldsymbol{\rho})$ into (24), $\hat{E}_{\mathrm{ho}}(x,y)$ can be recovered. $\hat{E}_{\mathrm{ho}}(x,y)$ is the reduced dimensionless transmitted field on an XY-plane near the hidden object, and its amplitude $\left|\hat{E}_{\mathrm{ho}}(x,y)\right|$ is the image of the hidden object. Finally, the image of the hidden object is successfully reconstructed.

Supplementary Note 2:

Calculation of the autocorrelation of hidden objects

In our experiment, the hidden object to be imaged is a transmission plate. The transmittance of the aperture of the transmission plate to the laser is 1, and the transmittance of other parts of the plate is close to 0. Because the size of the aperture on the transmission plate is in the order of mm, which is 10^3 times of the laser wavelength, the diffraction effect can be ignored. Based on the analysis above, the transmitted field of different hidden objects can be simulated.

The steps to simulate the transmitted field of a hidden object are as follows. First, an amplitude function $A_{\mathrm{ho}}(x,y)$ is generated. Then the center of the transmission plate is taken as the coordinate origin, and $A_{\mathrm{ho}}(x,y)$ is binarized according to the geometric characteristics

of the transmission plate. The rule of binarization is,

$$A_{\text{ho}}\left(x,y\right)=\begin{cases}1, & \left(x,y\right)\in \text{Hole area}\\ 0, & \left(x,y\right)\notin \text{Hole area}\end{cases} \tag{25}$$

Next, the phase of the transmitted field of the hidden object is simulated as a constant φ_{ho} that is independent of x or y. Finally, the simulated transmitted field of the hidden object is obtained,

$$E_{\text{ho}}\left(x,y\right)=A_{\text{ho}}\left(x,y\right)\cdot e^{i\varphi_{\text{ho}}} \tag{26}$$

Substituting the simulated $E_{\text{ho}}\left(x,y\right)$ into Eq. (21), (23), $C_{\text{ho}}\left(\Delta x,\Delta y\right)$ can be obtained. The simulated $A_{\text{ho}}\left(x,y\right)$ and $C_{\text{ho}}\left(\Delta x,\Delta y\right)$ of three different hidden objects are shown in the supplementary Figure 1.

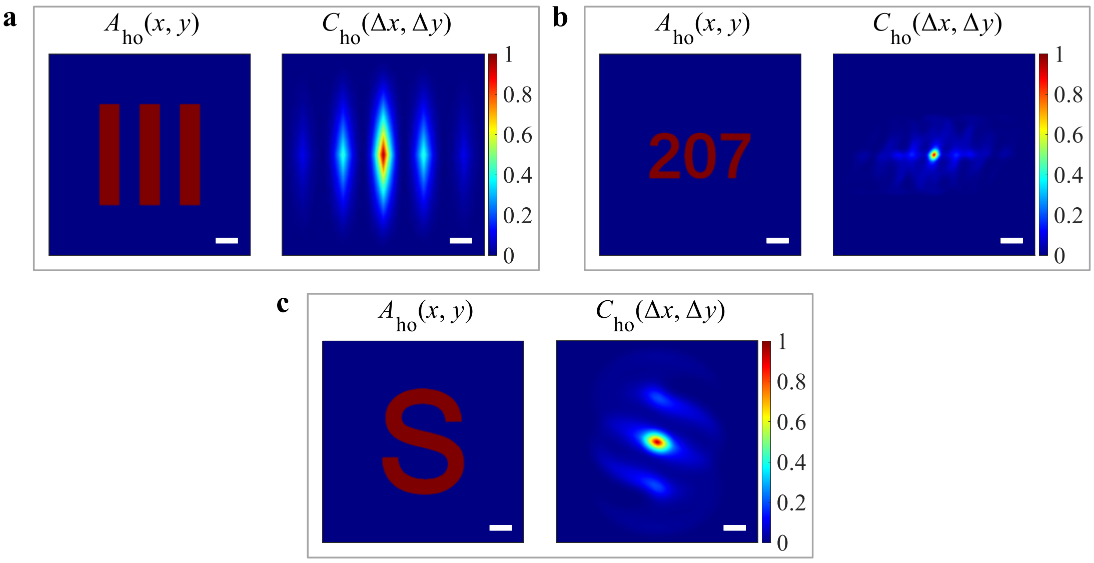

Supplementary Figure 1: Simulated $A_{\text{ho}}\left(x,y\right)$ and $C_{\text{ho}}\left(\Delta x,\Delta y\right)$ of three different hidden objects. a '|||'. **b** '207'. **c** 'S'. Scale bar: 1mm.

Supplementary Note 3:

Elimination of the adverse effect of ambient noise

Since the Eq.1 of the imaging relation is obtained without concerns for the noise, it is no longer valid when the speckle intensity images contain noise. In the condition of taking the noise into consideration, the reduced electric field E_{rd} of scattered light is expressed as,

$$E_{\text{rd}}\left(\mathbf{r}_{\text{c}};\mathbf{r}_{\text{o}}+\Delta\boldsymbol{\rho}\right)=E_{\text{rd-ho}}\left(\mathbf{r}_{\text{c}};\mathbf{r}_{\text{o}}+\Delta\boldsymbol{\rho}\right)+E_{\text{rd-b}}\left(\mathbf{r}_{\text{c}};t_0+\Delta t\right) \tag{27}$$

Here $E_{\text{rd-ho}}$ represents the scattering field induced by the transmitted field E_{ho} interacting with the random medium. $E_{\text{rd-b}}$ represents the time-varying background noise field. The position vector of the hidden object at time t_0 is $\mathbf{r}_{\text{o}}$, and the velocity vector of the hidden object is $\mathbf{v}$, then $\Delta\boldsymbol{\rho}=\mathbf{v}\Delta t$. Based on equation (27), the cross-correlation of E_{rd} can be expanded into four terms,

$$\begin{aligned}&\left\langle E_{\text{rd}}\left(\mathbf{r}_{\text{c}};\mathbf{r}_{\text{o}}\right)E_{\text{rd}}^{*}\left(\mathbf{r}_{\text{c}};\mathbf{r}_{\text{o}}+\Delta\boldsymbol{\rho}\right)\right\rangle_{\mathbf{r}_{\text{c}}}\\ =\;&\left\langle E_{\text{rd-ho}}\left(\mathbf{r}_{\text{c}};\mathbf{r}_{\text{o}}\right)E_{\text{rd-ho}}^{*}\left(\mathbf{r}_{\text{c}};\mathbf{r}_{\text{o}}+\Delta\boldsymbol{\rho}\right)+E_{\text{rd-b}}\left(\mathbf{r}_{\text{c}};t_0\right)E_{\text{rd-b}}^{*}\left(\mathbf{r}_{\text{c}};t_0+\Delta t\right)+\right.\\ &\left.E_{\text{rd-ho}}\left(\mathbf{r}_{\text{c}};\mathbf{r}_{\text{o}}\right)E_{\text{rd-b}}^{*}\left(\mathbf{r}_{\text{c}};t_0+\Delta t\right)+E_{\text{rd-b}}\left(\mathbf{r}_{\text{c}};t_0\right)E_{\text{rd-ho}}^{*}\left(\mathbf{r}_{\text{c}};\mathbf{r}_{\text{o}}+\Delta\boldsymbol{\rho}\right)\right\rangle_{\mathbf{r}_{\text{c}}}\end{aligned} \tag{28}$$

Since $E_{\text{rd-ho}}$ and $E_{\text{rd-b}}$ are independent of each other,

$$\left\langle E_{\text{rd-ho}}\left(\mathbf{r}_{\text{c}};\mathbf{r}_{\text{o}}\right)E_{\text{rd-b}}^{*}\left(\mathbf{r}_{\text{c}};t_0+\Delta t\right)+E_{\text{rd-b}}\left(\mathbf{r}_{\text{c}};t_0\right)E_{\text{rd-ho}}^{*}\left(\mathbf{r}_{\text{c}};\mathbf{r}_{\text{o}}+\Delta\boldsymbol{\rho}\right)\right\rangle_{\mathbf{r}_{\text{c}}}=0 \tag{29}$$

Substituting (29) into (28), then

$$\begin{aligned}&\left\langle E_{\text{rd}}\left(\mathbf{r}_{\text{c}};\mathbf{r}_{\text{o}}\right)E_{\text{rd}}^{*}\left(\mathbf{r}_{\text{c}};\mathbf{r}_{\text{o}}+\Delta\boldsymbol{\rho}\right)\right\rangle\\ =\;&\left\langle E_{\text{rd-ho}}\left(\mathbf{r}_{\text{c}};\mathbf{r}_{\text{o}}\right)E_{\text{rd-ho}}^{*}\left(\mathbf{r}_{\text{c}};\mathbf{r}_{\text{o}}+\Delta\boldsymbol{\rho}\right)\right\rangle_{\mathbf{r}_{\text{c}}}+\left\langle E_{\text{rd-b}}\left(\mathbf{r}_{\text{c}};t_0\right)E_{\text{rd-b}}^{*}\left(\mathbf{r}_{\text{c}};t_0+\Delta t\right)\right\rangle_{\mathbf{r}_{\text{c}}}\end{aligned} \tag{30}$$

Then substituting (30) into (7), we have

$$\begin{aligned}C_{\text{rd}}\left(\Delta\boldsymbol{\rho}\right)=\;&\frac{\left|\left\langle E_{\text{rd-ho}}\left(\mathbf{r}_{\text{c}};\mathbf{r}_{\text{o}}\right)E_{\text{rd-ho}}^{*}\left(\mathbf{r}_{\text{c}};\mathbf{r}_{\text{o}}+\Delta\boldsymbol{\rho}\right)\right\rangle_{\mathbf{r}_{\text{c}}}\right|^2+\left|\left\langle E_{\text{rd-b}}\left(\mathbf{r}_{\text{c}};t_0\right)E_{\text{rd-b}}^{*}\left(\mathbf{r}_{\text{c}};t_0+\Delta t\right)\right\rangle_{\mathbf{r}_{\text{c}}}\right|^2}{\left\langle\left|E_{\text{rd-ho}}\left(\mathbf{r}_{\text{c}};\mathbf{r}_{\text{o}}\right)\right|^2+\left|E_{\text{rd-b}}\left(\mathbf{r}_{\text{c}};t_0\right)\right|^2\right\rangle_{\mathbf{r}_{\text{c}}}^2}+\\ &\frac{\left\langle E_{\text{rd-ho}}\left(\mathbf{r}_{\text{c}};\mathbf{r}_{\text{o}}\right)E_{\text{rd-ho}}^{*}\left(\mathbf{r}_{\text{c}};\mathbf{r}_{\text{o}}+\Delta\boldsymbol{\rho}\right)\right\rangle_{\mathbf{r}_{\text{c}}}\left\langle E_{\text{rd-b}}^{*}\left(\mathbf{r}_{\text{c}};t_0\right)E_{\text{rd-b}}\left(\mathbf{r}_{\text{c}};t_0+\Delta t\right)\right\rangle_{\mathbf{r}_{\text{c}}}}{\left\langle\left|E_{\text{rd-ho}}\left(\mathbf{r}_{\text{c}};\mathbf{r}_{\text{o}}\right)\right|^2+\left|E_{\text{rd-b}}\left(\mathbf{r}_{\text{c}};t_0\right)\right|^2\right\rangle_{\mathbf{r}_{\text{c}}}^2}+\\ &\frac{\left\langle E_{\text{rd-ho}}^{*}\left(\mathbf{r}_{\text{c}};\mathbf{r}_{\text{o}}\right)E_{\text{rd-ho}}\left(\mathbf{r}_{\text{c}};\mathbf{r}_{\text{o}}+\Delta\boldsymbol{\rho}\right)\right\rangle_{\mathbf{r}_{\text{c}}}\left\langle E_{\text{rd-b}}\left(\mathbf{r}_{\text{c}};t_0\right)E_{\text{rd-b}}^{*}\left(\mathbf{r}_{\text{c}};t_0+\Delta t\right)\right\rangle_{\mathbf{r}_{\text{c}}}}{\left\langle\left|E_{\text{rd-ho}}\left(\mathbf{r}_{\text{c}};\mathbf{r}_{\text{o}}\right)\right|^2+\left|E_{\text{rd-b}}\left(\mathbf{r}_{\text{c}};t_0\right)\right|^2\right\rangle_{\mathbf{r}_{\text{c}}}^2}\end{aligned} \tag{31}$$

It can be found from Eq.(31) that $C_{\text{rd}}\left(\Delta\boldsymbol{\rho}\right)$ contains both the correlation of noise and of transmitted field of the hidden object. When the noise evolves rapidly with time, its time correlation can be seen as a δ function, therefore,

$$\left\langle E_{\text{rd-b}}\left(\mathbf{r}_{\text{c}};t_0\right)E_{\text{rd-b}}^{*}\left(\mathbf{r}_{\text{c}};t_0+\Delta t\right)\right\rangle_{\mathbf{r}_{\text{c}}}=\begin{cases}\left\langle I_{\text{rd-b}}\left(\mathbf{r}_{\text{c}};t_0\right)\right\rangle_{\mathbf{r}_{\text{c}}}, & \Delta t=0\\ 0, & \Delta t>0\end{cases} \tag{32}$$

Substituting (32) into (31),

$$C_{\mathrm{rd}}(\Delta\boldsymbol{\rho})=\begin{cases}1 & , \ |\Delta\boldsymbol{\rho}|=0\\ \dfrac{\left|\left\langle E_{\text{rd-ho}}(\mathbf{r}_{\mathrm{c}};\mathbf{r}_{\mathrm{o}})E^{*}_{\text{rd-ho}}(\mathbf{r}_{\mathrm{c}};\mathbf{r}_{\mathrm{o}}+\Delta\boldsymbol{\rho})\right\rangle_{\mathbf{r}_{\mathrm{c}}}\right|^{2}}{\left\langle\left|E_{\text{rd-ho}}(\mathbf{r}_{\mathrm{c}};\mathbf{r}_{\mathrm{o}})\right|^{2}+\left|E_{\text{rd-b}}(\mathbf{r}_{\mathrm{c}};t_{0})\right|^{2}\right\rangle^{2}_{\mathbf{r}_{\mathrm{c}}}} & , \ |\Delta\boldsymbol{\rho}|>0\end{cases} \tag{33}$$

It can be found from Eq.(33) that $C_{\mathrm{rd}}(\Delta\boldsymbol{\rho})$ is a piecewise function. When $|\Delta\boldsymbol{\rho}|$ increases gradually from 0, the noise causes $C_{\mathrm{rd}}(\Delta\boldsymbol{\rho})$ to drop sharply from 1 to $\left|\left\langle I_{\text{rd-ho}}(\mathbf{r}_{\mathrm{c}};\mathbf{r}_{\mathrm{o}})\right\rangle_{\mathbf{r}_{\mathrm{c}}}/\left\langle I_{\mathrm{rd}}(\mathbf{r}_{\mathrm{c}};\mathbf{r}_{\mathrm{o}})\right\rangle_{\mathbf{r}_{\mathrm{c}}}\right|^{2}$, it deviates significantly from $C_{\mathrm{ho}}(\Delta\boldsymbol{\rho})$. In the Supplementary Figure 2, C_{rd} calculated from the experimental data is compared with C_{ho}, which confirms the sudden drop behavior and shows the significant deviation between the two correlations.

In order to image hidden objects, the adverse effect of noise on $C_{\mathrm{rd}}(\Delta\boldsymbol{\rho})$ must be eliminated. We denote the cross-correlation of speckle intensity images with the noise elimination as $\mathcal{C}_{\mathrm{rd}}(\Delta\boldsymbol{\rho})$,

$$\mathcal{C}_{\mathrm{rd}}(\Delta\boldsymbol{\rho})=\frac{\left|\left\langle E_{\text{rd-ho}}(\mathbf{r}_{\mathrm{c}};\mathbf{r}_{\mathrm{o}})E^{*}_{\text{rd-ho}}(\mathbf{r}_{\mathrm{c}};\mathbf{r}_{\mathrm{o}}+\Delta\boldsymbol{\rho})\right\rangle_{\mathbf{r}_{\mathrm{c}}}\right|^{2}}{\left\langle I_{\text{rd-ho}}(\mathbf{r}_{\mathrm{c}};\mathbf{r}_{\mathrm{o}})\right\rangle^{2}_{\mathbf{r}_{\mathrm{c}}}} \tag{34}$$

According to **Supplementary Note 1**, $\mathcal{C}_{\mathrm{rd}}(\Delta\boldsymbol{\rho})\approx C_{\mathrm{ho}}(\Delta\boldsymbol{\rho})$, $\mathcal{C}_{\mathrm{rd}}(\Delta\boldsymbol{\rho})$, therefore, can be used to reconstruct the image of hidden objects. In order to recover $\mathcal{C}_{\mathrm{rd}}(\Delta\boldsymbol{\rho})$, $\left|\left\langle E_{\text{rd-ho}}(\mathbf{r}_{\mathrm{c}};\mathbf{r}_{\mathrm{o}})E^{*}_{\text{rd-ho}}(\mathbf{r}_{\mathrm{c}};\mathbf{r}_{\mathrm{o}}+\Delta\boldsymbol{\rho})\right\rangle_{\mathbf{r}_{\mathrm{c}}}\right|^{2}$ is extracted from $C_{\mathrm{rd}}(\Delta\boldsymbol{\rho})$. By inverting Eq. (33), we have,

$$\left|\left\langle E_{\text{rd-ho}}(\mathbf{r}_{\mathrm{c}};\mathbf{r}_{\mathrm{o}})E^{*}_{\text{rd-ho}}(\mathbf{r}_{\mathrm{c}};\mathbf{r}_{\mathrm{o}}+\Delta\boldsymbol{\rho})\right\rangle_{\mathbf{r}_{\mathrm{c}}}\right|^{2}=\begin{cases}\left\langle I_{\text{rd-ho}}(\mathbf{r}_{\mathrm{c}};\mathbf{r}_{\mathrm{o}})\right\rangle^{2}_{\mathbf{r}_{\mathrm{c}}} & , \ |\Delta\boldsymbol{\rho}|=0\\ C_{\mathrm{rd}}(\Delta\boldsymbol{\rho})\cdot\left\langle I_{\mathrm{rd}}(\mathbf{r}_{\mathrm{c}};\mathbf{r}_{\mathrm{o}})\right\rangle^{2}_{\mathbf{r}_{\mathrm{c}}} & , \ |\Delta\boldsymbol{\rho}|>0\end{cases} \tag{35}$$

From equation (35), it can be found that all values of $\left|\left\langle E_{\text{rd-ho}}(\mathbf{r}_{\mathrm{c}};\mathbf{r}_{\mathrm{o}})E^{*}_{\text{rd-ho}}(\mathbf{r}_{\mathrm{c}};\mathbf{r}_{\mathrm{o}}+\Delta\boldsymbol{\rho})\right\rangle_{\mathbf{r}_{\mathrm{c}}}\right|^{2}$ are known except for $\Delta\boldsymbol{\rho}=0$. Therefore, $\left\langle I_{\text{rd-ho}}(\mathbf{r}_{\mathrm{c}};\mathbf{r}_{\mathrm{o}})\right\rangle^{2}_{\mathbf{r}_{\mathrm{c}}}$ can be extrapolated from the known

values of $\left|\left\langle E_{\text{rd-ho}}\left(\mathbf{r}_c;\mathbf{r}_o\right)E^*_{\text{rd-ho}}\left(\mathbf{r}_c;\mathbf{r}_o+\Delta\boldsymbol{\rho}\right)\right\rangle_{\mathbf{r}_c}\right|^2$. Then $C_{\text{rd}}\left(\Delta\boldsymbol{\rho}\right)$ can be recovered with equation (34).

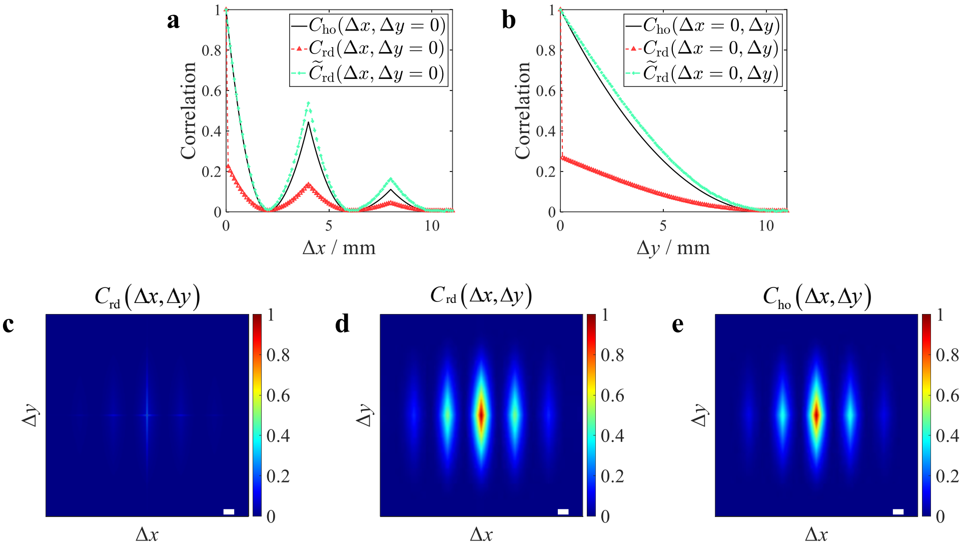

Supplementary Figure 2: A comparison of C_{ho}, C_{rd} and C_{rd}. a C_{ho}, C_{rd} and C_{rd} vs Δx. **b** C_{ho}, C_{rd} and C_{rd} vs Δy. **c** $C_{\text{rd}}\left(\Delta x, \Delta y\right)$. **d** $C_{\text{rd}}\left(\Delta x, \Delta y\right)$. **e** $C_{\text{ho}}\left(\Delta x, \Delta y\right)$. Scale bar: 1mm.

We use quadratic polynomial to extrapolate $\left\langle I_{\text{rd-ho}}\left(\mathbf{r}_c;\mathbf{r}_o\right)\right\rangle^2_{\mathbf{r}_c}$. First, data points of $\left|\left\langle E_{\text{rd-ho}}\left(\mathbf{r}_c;\mathbf{r}_o\right)E^*_{\text{rd-ho}}\left(\mathbf{r}_c;\mathbf{r}_o+\Delta\boldsymbol{\rho}\right)\right\rangle_{\mathbf{r}_c}\right|^2$ in a monotonic decreasing region $\left(0<\Delta x\le l_x, 0<\Delta y\le l_y\right)$ near $\left|\Delta\boldsymbol{\rho}\right|=0$ are chosen. Then these data points are substituted into the following quadratic polynomials,

$$C_{\text{rd}}\left(\Delta x,\Delta y\right)\cdot\left\langle I_{\text{rd}}\left(\mathbf{r}_c;\mathbf{r}_o\right)\right\rangle^2_{\mathbf{r}_c}=\left\langle I_{\text{rd-ho}}\left(\mathbf{r}_c;\mathbf{r}_o\right)\right\rangle^2_{\mathbf{r}_c}+k_1\left(\Delta x+\Delta y\right)+k_2\left(\Delta x^2+\Delta y^2\right) \tag{36}$$

Since the expression contains three unknown coefficients $\left\langle I_{\text{rd-ho}}\left(\mathbf{r}_c;\mathbf{r}_o\right)\right\rangle^2_{\mathbf{r}_c}$, k_1 and k_2, at least three data points are required to solve these coefficients. We select three data points on x-axis, y-axis and 45 ° direction respectively, and substitute them into Eq. (36). Three groups of coefficients $\left\langle I_{\text{rd-ho}}\left(\mathbf{r}_c;\mathbf{r}_o\right)\right\rangle^2_{\mathbf{r}_c}$, k_1 and k_2 are solved. Then three values of $\left\langle I_{\text{rd-ho}}\left(\mathbf{r}_c;\mathbf{r}_o\right)\right\rangle^2_{\mathbf{r}_c}$ are statistically averaged and substituted into Eq. (34) to obtain $C_{\text{rd}}\left(\Delta\boldsymbol{\rho}\right)$.

The recovered C_{rd} is also drawn in Supplementary Figure 2. The good consistency between C_{rd} and C_{ho} indicates that the noise is successfully eliminated, and confirms the

adaptability of the RSESIC method in an environment with strong noise.

Supplementary Note 4:

Experimental comparison of reconstruction imaging between the RSESIC and SSIC method in a random corridor

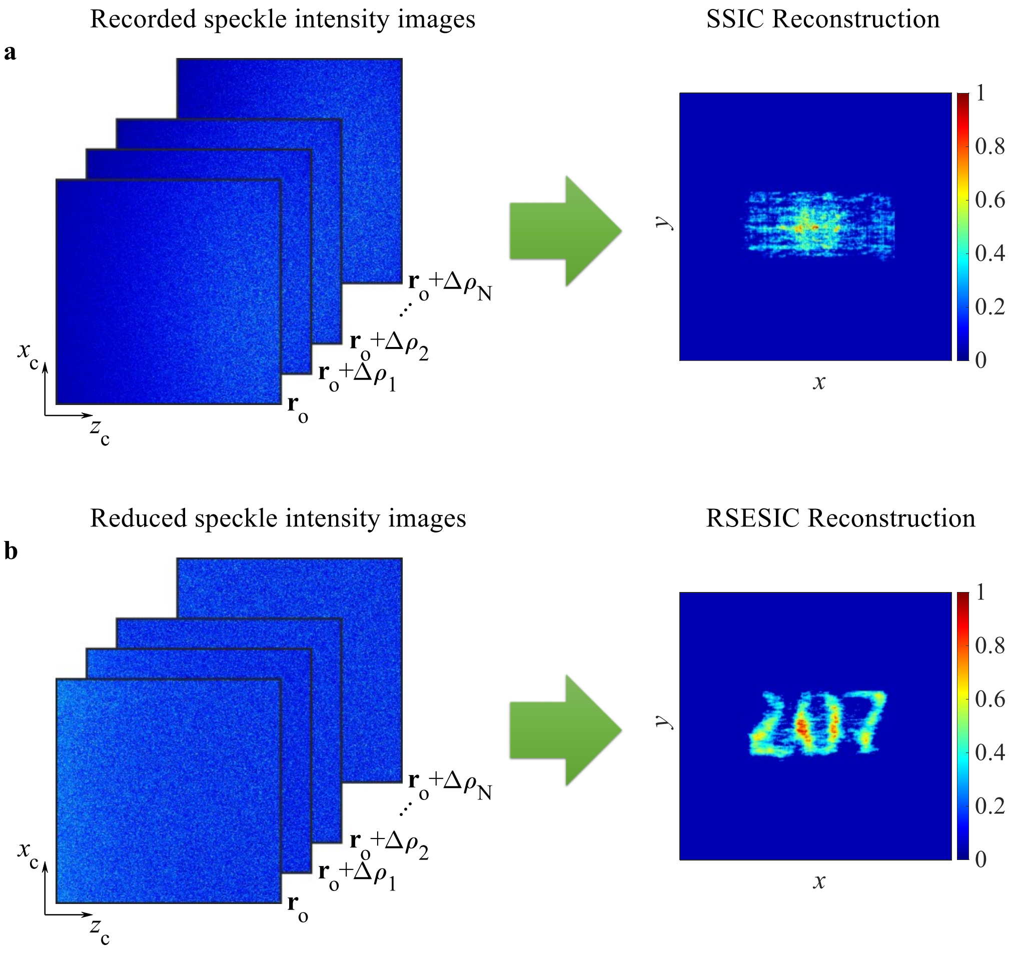

Supplementary Figure 3: Comparison of reconstruction imaging between the RSESIC and SSIC method in a random corridor. a Raw speckle intensity images $I_s(\mathbf{r}_c;\mathbf{r}_o+\Delta\rho)$ and hidden object's image reconstructed by the SSIC method. **b** Reduced speckle intensity images $I_{rd}(\mathbf{r}_c;\mathbf{r}_o+\Delta\rho)$ and hidden object's image reconstructed by the RSESIC method.

Under the computational imaging condition in Fig. 1a of the main text, the imaging ability between the RSESIC and previous SSIC method is compared experimentally. The previous SSIC method does not apply the subspace reduction technique used in the RSESIC method, but rather directly uses the speckle intensity images $I_s(\mathbf{r}_c;\mathbf{r}_o+\Delta\rho)$ taken by a camera to reconstruct the hidden object. So the SSIC method cannot solve the reconstruction problem in

random corridors. The failure of the SSIC method has been experimentally verified, as shown in Supplementary Figure 3a. Here, the image reconstructed by the SSIC method cannot reveal any features of the hidden object. By comparison, the RSESIC method successfully restores the image of hidden object from reduced speckle intensity images $I_{\mathrm{rd}}\left(\mathbf{r}_{\mathrm{c}};\mathbf{r}_{\mathrm{o}}+\Delta\rho\right)$, as shown in Supplementary Figure 3b.

Supplementary Note 5:

Experimental comparison between the RSESIC and SSIC method for imaging through a thick random medium

The RSESIC method, in a sense, is a speedup over previous work and allows to see through thicker random media. In order to confirm this, we performed experiments of imaging a hidden object behind random media with different thicknesses and compare the imaging quality.

Supplementary Figure 4a shows the schematic diagram of the imaging system, and the thickness of the diffuser is denoted as L. Supplementary Figure 4b shows the hidden object that is a metal plate with three slits. Milky acrylic sheets with thicknesses $L=10mm$, $30mm$ and $50mm$ were used in the experiment as the random media. The photographs shown in Supplementary Figure 4c reveal the strong scattering characteristics of these milky white acrylic sheets. In Supplementary Figure 4d, the hidden object images reconstructed by the RSESIC and SSIC method are compared. It can be clearly seen in Supplementary Figure 4d that the SSIC method fails as $L>30mm$, but the RSESIC method can still work when $L=50mm$. This result proves the advantage of the RSESIC imaging method for a thick random medium.

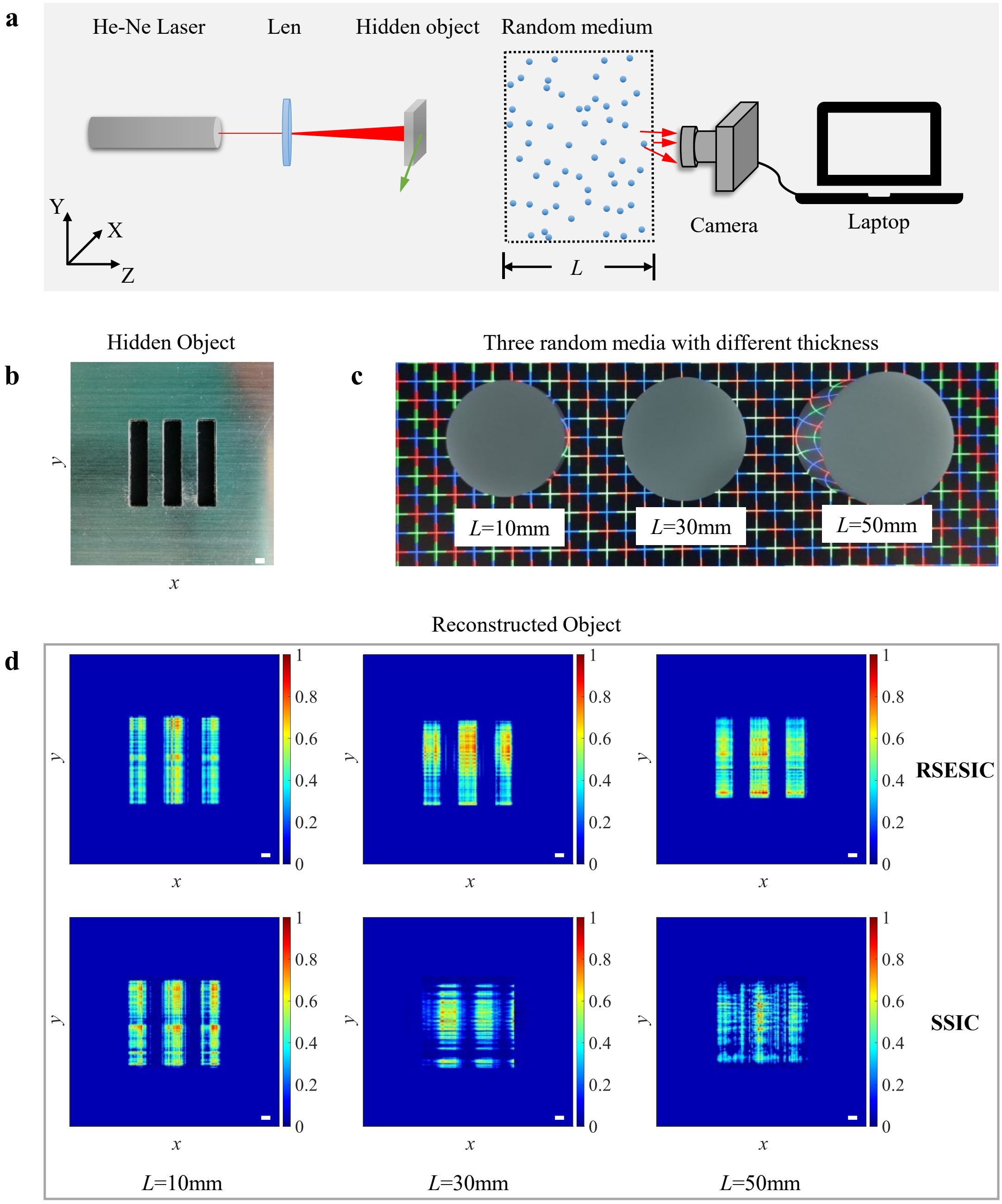

Supplementary Figure 4: Experimental comparison of imaging a hidden object obscured by random media with different thicknesses. **a** Schematic diagram of the experimental system. **b** Actual photograph of the hidden object. **c** Photographs of random media with different thicknesses ($L = 10$, 30, 50 mm). **d** Comparison of reconstructed images between two methods, RSESIC and SSIC.

Supplementary Note 6:

Imaging a hidden object with reflected laser speckle intensity images

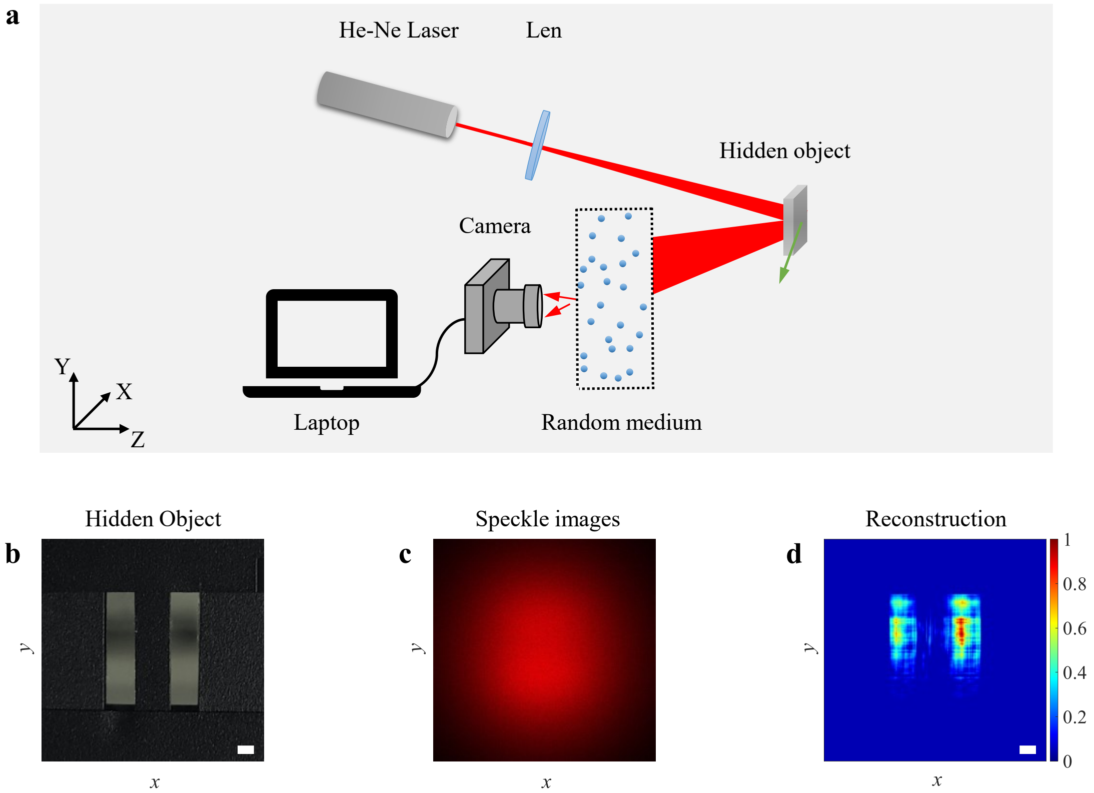

Supplementary Figure 5: Imaging a hidden object with reflected laser speckle intensity images. a Schematic diagram. **b** Actual photograph of the hidden object. **c** Reflected laser speckle intensity image. **d** Hidden object image reconstructed by the RSESIC method

Supplementary Figure 5 shows the experimental setup and results of imaging a hidden object with reflected laser speckle. Because there are no a pulsed laser and fast camera in our lab, the expanded laser directly shines on the hidden object in order to avoid the adverse effect of direct back reflections by a random medium, as shown in Supplementary Figure 5a. The RSESIC method can reconstruct the image of a hidden object by using a He-Ne laser and an ordinary camera. Supplementary Figure 5b shows the photo of the hidden object. The reflected laser speckle intensity image shown in Supplementary Figure 5c confirms that the object is completely obscured by the random medium. Supplementary Figure 5d demonstrates the hidden object image reconstructed by the RSESIC method. The good agreement between Supplementary Figure 5b and Supplementary Figure 5d shows that the RSESIC method is suitable for the reflection imaging system.

Supplementary Note 7:

Imaging an object hidden in a two-pass scattering system

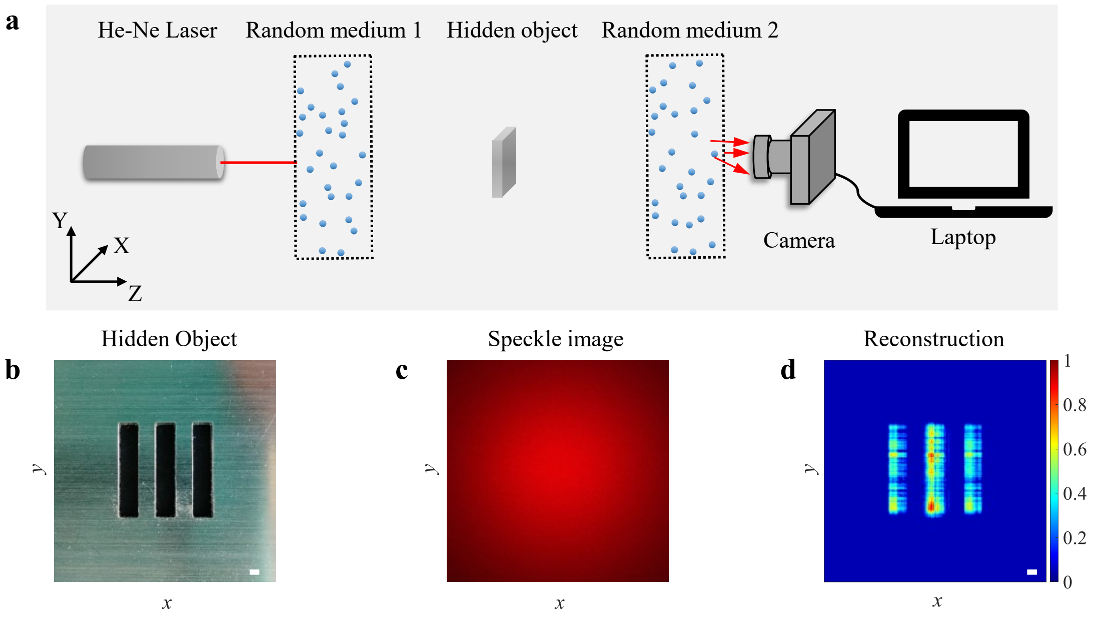

Supplementary Figure 6: Imaging an object hidden in a two-pass scattering system. a Schematic diagram. **b** Actual photo of the hidden object. **c** Transmitted laser speckle intensity image. **d** Hidden object image reconstructed by the RSESIC method

Supplementary Figure 6 exhibits the imaging scenario and results for a two-pass scattering system. Supplementary Figure 6a shows the imaging scenario, in which the object is sandwiched between two opaque, random media. Supplementary Figure 6b shows the photo of the hidden object. The transmitted laser speckle intensity image shown in Supplementary Figure 6c confirms that the object is completely obscured by the random medium. Supplementary Figure 6d demonstrates the hidden object image reconstructed by the RSESIC method. The reconstructed image (Supplementary Figure 6d) is consistent with the actual one of hidden object (Supplementary Figure 6b), showing that the RSESIC method is still valid for a two-pass scattering system.

References

1. Reed IS. On a moment theorem for complex Gaussian processes. *IRE Transactions on Information Theory* **8**, 194-195 (1962).

2. Goodman JW. *Speckle Phenomena in Optics: Theory and Applications*. Roberts & Co (2007).

3. Martin OJF, Girard C, Dereux A. Generalized Field Propagator for Electromagnetic Scattering and Light Confinement. *Physical Review Letters* **74**, 526-529 (1995).

4. Goodman J. *Introduction To Fourier Optics*, 2 edn. The McGraw-Hill Companies (1996).

5. Fienup JR. Reconstruction of a complex-valued object from the modulus of its Fourier transform using a support constraint. *J Opt Soc Am A* **4**, 118-123 (1987).

6. Meihua L, Dajiang L, Wenqi H, Giancarlo P, Wolfgang O, Xiang P. Improving reconstruction of speckle correlation imaging by using a modified phase retrieval algorithm with the number of nonzero-pixels constraint. *Appl Opt* **58**, 473-478 (2019).
